# The association between procalcitonin and acute kidney injury in patients stung by wasps

**DOI:** 10.3389/fphys.2023.1199063

**Published:** 2023-08-28

**Authors:** Xuepeng Zhang, Yunxia Feng, Kai Wang, Tong Qiu, Jiangyuan Zhou, Guowei Che, Siyuan Chen, Yi Ji

**Affiliations:** ^1^ Department of Pediatric Surgery, West China Hospital of Sichuan University, Chengdu, China; ^2^ Department of Critical Care Medicine, West China Hospital of Sichuan University, Chengdu, China; ^3^ Department of Nephrology, Mianyang Central Hospital, University of Electronic Science and Technology of China, Mianyang, China; ^4^ Department of Thoracic Surgery, West China Hospital of Sichuan University, Chengdu, China

**Keywords:** wasp sting, procalcitonin, acute kidney injury, association, prediction

## Abstract

**Introduction:** The aim of this study was to investigate the status of serum procalcitonin (PCT) in patients stung by wasps and evaluate the association between PCT levels and acute kidney injury (AKI).

**Methods:** Patients stung by wasps admitted to two tertiary hospitals between January 2017 and December 2020 were screened for enrollment. We evaluated serum PCT levels on admission in patients stung by wasps. The patients were divided into an AKI group and a non-AKI group. A logistic regression model was used to analyze the association between PCT status and AKI. The performance of PCT concentrations in predicting the occurrence of AKI was evaluated by the area under the receiver operating characteristic curve (AUROC).

**Results:** A total of 138 patients were enrolled, and 66 patients suffered AKI. PCT levels were elevated in 78.99% of patients stung by wasps. Nearly half of the patients (47.83%) developed AKI. PCT levels were correlated with creatinine levels on admission (r = 0.787, 95% CI: 0.713–0.844). PCT levels in patients with AKI were higher than those in patients without AKI (*p* < 0.001). After adjustment for covariates, PCT levels on admission were independently associated with AKI (OR: 1.575, 95% CI: 1.071–2.317, *p* = 0.021). The AUROC of PCT levels on admission was 0.837 (95% CI, 0.771–0.902, *p* < 0.001). A PCT level of 0.57 μg/L was the cutoff for maximizing the Youden index; the specificity was 79.45%, and the sensitivity was 73.43%.

**Conclusion:** Serum PCT levels may be a potential biomarker of AKI in patients stung by wasps.

## 1 Introduction

Wasp sting is a common public health problem in tropical countries, particularly in rural areas of eastern and southern Asia (Bhuiyan et al., 2019). Recently, wasp sting is becoming an emerging problem in other countries such as in Europe and America, because wasp incursions are increasing in the global change scenario (Herrera et al., 2020). The wasp sting could lead to life-threatening multiple organ dysfunction including intravascular hemolysis, rhabdomyolysis, clotting abnormality, acute kidney injury (AKI), and liver dysfunction (Ambarsari et al., 2019). AKI is the prominent organ dysfunction occurring in more than half of the patients (Yuan et al., 2020). Notably, the development of AKI significantly increases the risk of mortality in patients with wasp sting (Tang et al., 2022). The mortality of patients with AKI was highly as 50% (Vikrant and Parashar, 2017).

Until now, the mechanism of wasp sting-induced AKI is not clear. Intravascular hemolysis and rhabdomyolysis had been considered as the causes of wasp sting-induced AKI (Thiruventhiran et al., 1999). However, recent studies have revealed that the inflammatory response plays an important role in the development of AKI among patients with wasp sting. The inflammatory response in the kidney mediated by the stimulator of interferon genes (STING) signaling pathway prompts wasp venom-induced kidney injury (Lv et al., 2023). Another study revealed that phospholipase A2 (PLA2), a component of wasp venom, could induce renal tubular epithelial cell apoptosis by the complement mediated mitochondrial apoptosis pathway to cause AKI via activation of the TNF-α/NF-κB signaling pathway (Tang et al., 2023).

Currently, treatments for AKI are still disappointing. Early identification of patients at high risk of AKI is very important in the overall management of AKI. However, there are few studies on the prediction of AKI among people who are stung by wasps. Serum procalcitonin (PCT), a quick-response biomarker of inflammation, is readily available in most hospitals. We hypothesize that PCT may be helpful in predicting AKI caused by wasp stings. In the present study, we investigated PCT levels in patients stung by wasps and evaluated the association between the PCT levels and development of AKI in patients with wasp stings.

## 2 Materials and methods

This was a prospective observational study conducted in two tertiary hospitals, located in western China. The two hospitals serve a population of more than 30 million people. All adult wasp sting patients admitted to the two hospitals from January 2017 to December 2020 were screened for enrollment. The Ethics Committee of West China Hospital of Sichuan University approved this study. Patients included in the study all provided informed consent. All procedures were in compliance with the Declaration of Helsinki. The criteria for exclusion were as follows: age <18 years; patients who had been diagnosed with AKI in other hospitals before referral; patients who had a history of chronic kidney disease (CKD); and refusal to participate in the study.

Data including demographic data (age, sex, *etc.*) were collected on admission. Medical history of hypertension, diabetes and CKD was also collected on admission. Blood samples will be collected upon admission for laboratory tests, including hemoglobin (Hb), serum creatinine, white blood cell (WBC) count, and PCT. The patients will be followed up until death during the hospital stay or hospital discharge. Serum creatinine values and urine information within 7 days following admission were collected for the identification of AKI. Clinical characteristics, including the development of AKI during hospital stay, length of hospital stay, hospital mortality, ICU admission, *etc.*, were also collected. A PCT level higher than 0.05 μg/L was considered to indicate an increased level.

The primary outcome of this study was the development of AKI during the entire hospital stay. AKI is defined as an abrupt decrease in kidney function within 7 days, which is determined by a decrease in urine or an increase in serum creatinine within a specific time, in accordance with the Kidney Disease: Improving Global Outcomes (KDIGO) clinical practice guidelines (Kellum et al., 2012). The classification for AKI is as follows: stage 1, increase in serum creatinine level to ≥26.5 μmol/L within 48 h or increase in serum creatinine level by 1.5–1.9 times the baseline within 7 days; stage 2, increase in serum creatinine level by 2.0–2.9 times the baseline within 7 days; and stage 3, increase in serum creatinine level by ≥ 3.0 times the baseline, or increase in serum creatinine level to ≥353.6 μmol/L with either increase to ≥26.5 μmol/L within 48 h or increase ≥50% from baseline within 7 days, or initiation of renal replacement therapy. The baseline serum creatinine concentration was measured at the time of admission.

Statistical analyses were conducted by using SPSS 22.0 for Windows (SPSS Inc., Chicago, IL, United States). Continuous variables are presented as the means ± standard deviations (SDs) or medians with 25% and 75% quartiles (interquartile ranges, IQRs) as appropriate and were analyzed by using Student’s t-test or a nonparametric test (Mann‒Whitney *U* test). Categorical variables were expressed as counts or proportions and analyzed by using the chi-squared test or Fisher’s exact test. Correlation was evaluated by Pearson correlation or Spearman correlation, depending on the distribution of variables. Logistic regression analysis was used to evaluate the association between PCT and AKI. Variables with *p* < 0.10 in the univariate analysis were analyzed in the multivariable analysis. We analyzed the performance of PCT for predicting AKI by using receiver operating characteristic (ROC) curves. The Youden index was used to determine the optimal PCT value cutoff for AKI.

## 3 Results

There were a total of 147 wasp sting patients during the study period. Nine patients were excluded: 5 patients were younger than 18 years, 2 had CKD and 2 had AKI on admission. One hundred thirty-eight patients were ultimately enrolled. The mean age of the population was 62.34 ± 12.93 years (Table 1). More than half of the patients were men (53.62%). The median (IQR) PCT level was 1.30 (0.46, 3.89) μg/L. One hundred nine patients (78.99%) had elevated PCT concentrations. The median length of time between wasp sting and hospitalization was 7.0 h (IQR, 5.0–10.50 h). The median length of hospital stay was 14.0 (6.0, 26.0) days. There were 66 (47.83%) patients who developed AKI during their hospital stay: 21 suffered stage 1 AKI, 5 patients had stage 2 AKI and 40 had stage 3 AKI. The mortality of the study population was 6.52%.

**TABLE 1:** Characteristics of 138 patients with wasp stings.

Characteristics	Value
Age, year	62.34 ± 12.93
Male, n (%)	74 (53.62%)
PCT, μg/L	1.30 (0.46, 3.89)
PCT >0.05 μg/L, n (%)	109 (78.99%)
Hb, g/L	119.72 ± 22.97
WBC, ×10^9^/L	21.25 ± 7.15
Time elapse from bite to hospital, hours	7.0 (5.0, 10.50)
Underlying disease, n (%)	
Hypertension	22 (15.94%)
Diabetes	6 (4.35%)
RRT, n (%)	32 (23.19%)
Oliguria, n (%)	12 (8.70%)
Anuria, n (%)	22 (15.94%)
Time point of the highest creatinine, days	5 (2, 9)
Length of hospital stay, days	14.0 (6.0, 26.0)
AKI, n (%)	66 (47.83%)
AKI stage 1	21 (15.21%)
AKI stage 2	5 (3.62%)
AKI stage 3	40 (28.99%)
Death, n (%)	9 (6.52%)

Abbreviations: PCT, procalcitonin; Hb, Hemoglobin; WBC, white blood cell counts; RRT, renal replacement therapy; AKI, acute kidney injury.

Data are presented as the mean ± standard deviation, median (interquartile range), or number (percentage).

**FIGURE 1:**
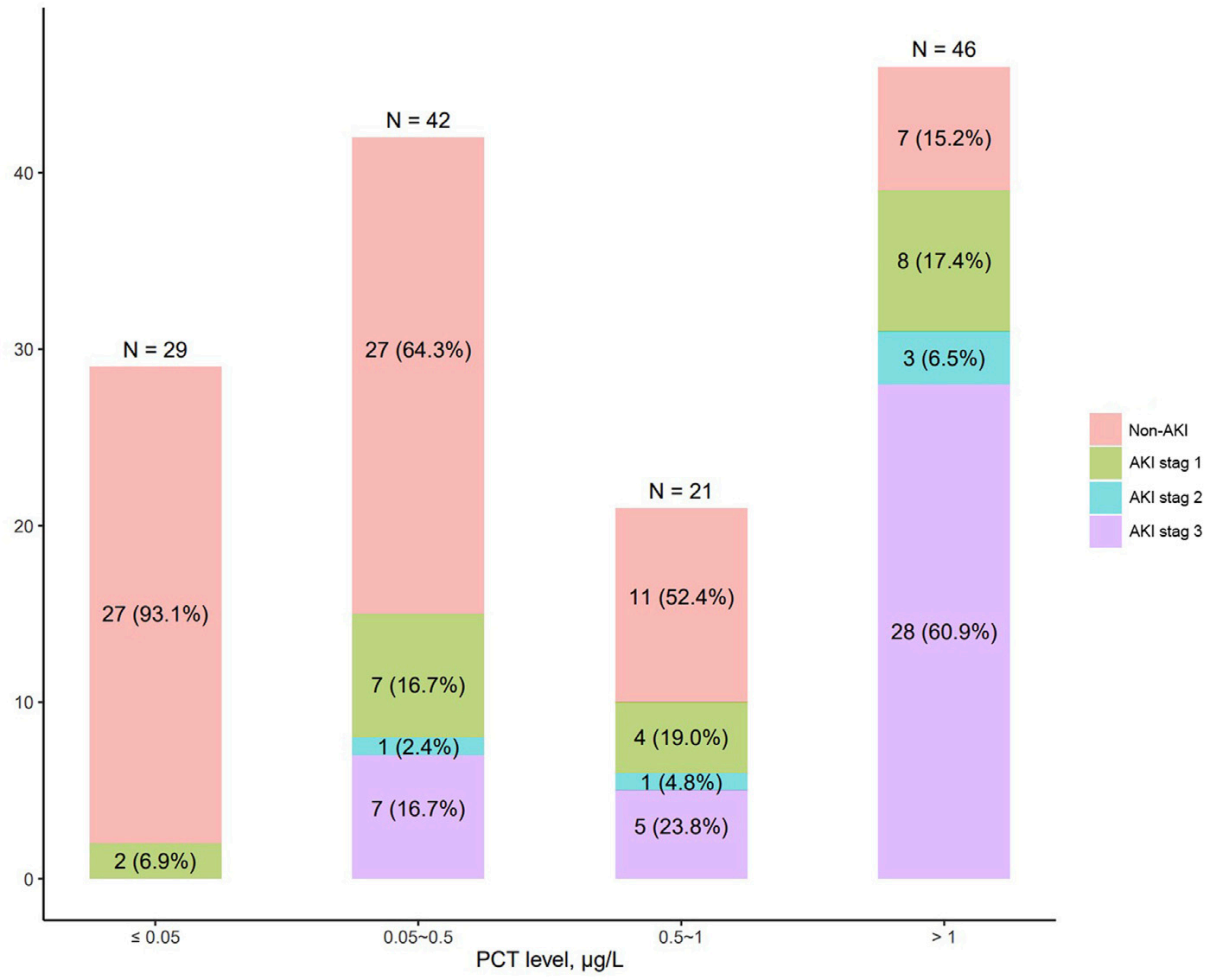
Distribution of AKI in patients with different PCT levels.

**FIGURE 2:**
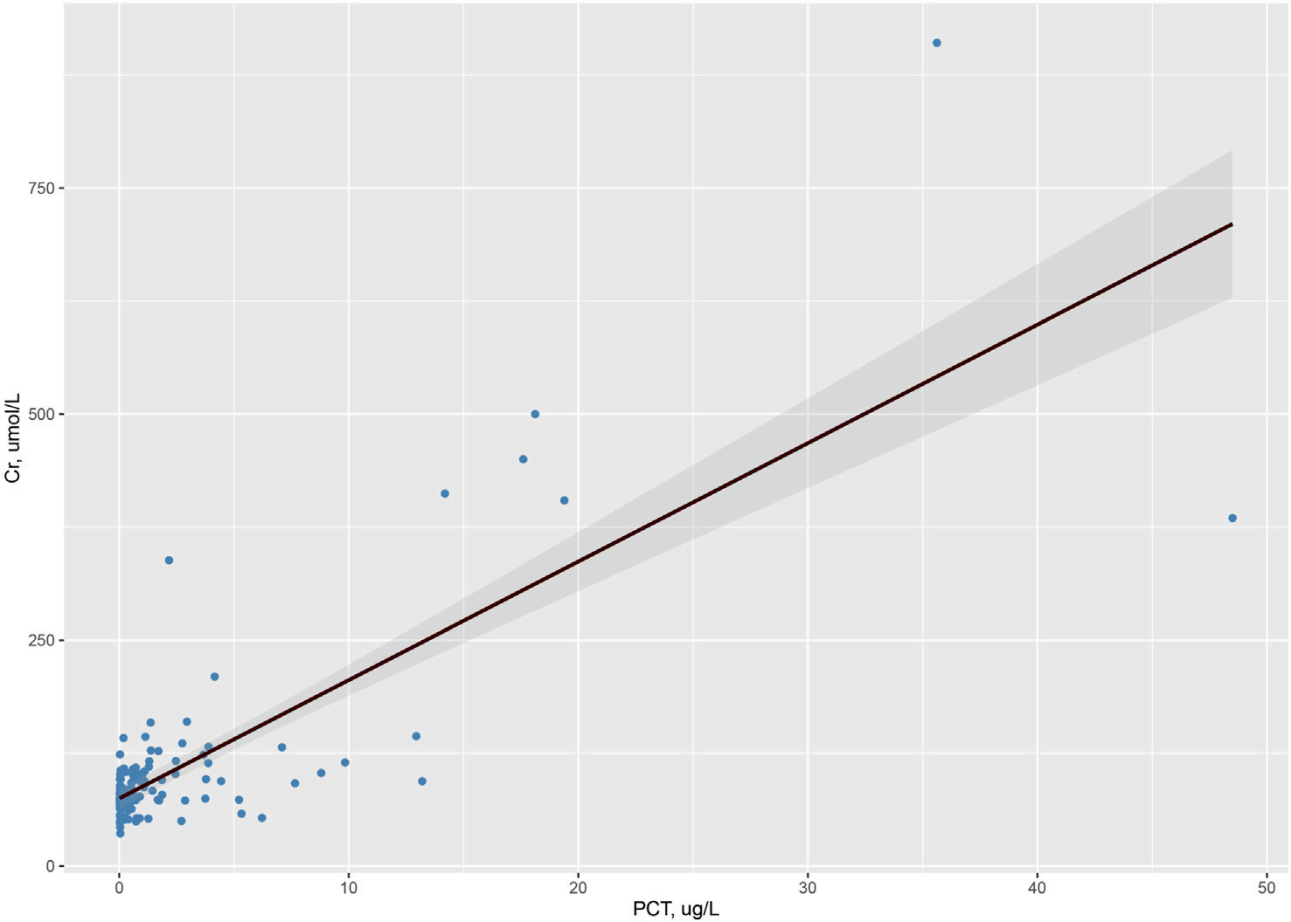
Correlation between PCT levels and creatinine levels on admission (r = 0.787, 95% CI 0.713–0.844, *p* < 0.001).

Figure 1 shows the distribution of AKI in patients with different PCT levels. The incidence of AKI increased with increasing serum PCT levels. The rate of AKI was 6.9% in patients with normal PCT levels, 35.7% in patients with PCT levels of 0.05–0.5 μg/L, 47.6% in patients with PCT levels of 0.5–1.0 μg/L, 84.8% in patients with PCT levels of >1 μg/L. In addition, the incidence of stage 3 AKI was also highest in patients with PCT levels of >1 μg/L, with a rate of 60.9%. Figure 2 shows the correlation between PCT levels and creatinine levels on admission. PCT levels were strongly correlated with creatinine levels, with r = 0.787 (95% CI 0.713–0.844, *p* < 0.001).

A comparison of characteristics on admission between the AKI group and the non-AKI group is shown in Table 2. The AKI group was significantly older than the non-AKI group (*p* = 0.002). The AKI group had higher serum PCT, WBC, and creatinine levels and lower hemoglobin levels. The elapsed time from sting to hospital admission was longer in the AKI group, but the difference was not significant (*p* = 0.067). The distributions of sex, hypertension, and diabetes were similar in the two groups. There were higher rates of ICU admission, ventilation, and death in the AKI group.

**TABLE 2:** Comparison of the characteristics on admission between AKI group and non-AKI group.

	AKI N = 66	Non-AKI N = 72	*p*-Value
Age, y	62.34 ± 12.93	54.88 ± 14.50	0.002^a^
Male, n (%)	29 (43.94%)	45 (62.50%)	0.045^b^
PCT, μg/L	1.30 (0.46, 3.89)	0.10 (0.04, 0.53)	0.000^c^
Creatinine, μmol/L	102.90 (79.60, 131.75)	73.40 (63.60, 82.05)	0.000^c^
WBC, ×10^9^/L	21.25 ± 7.15	14.99 ± 6.96	0.000^a^
Hb, g/L	119.72 ± 22.97	140.70 ± 18.04	0.000^a^ ^c^
Time elapse from bite to hospital, hours	7.0 (5.0, 10.0)	6.0 (2.0, 12.0)	0.067^d^
Hypertension, n (%)	8 (12.12%)	14 (19.44%)	0.271^b^
Diabetes, n (%)	4 (6.06%)	2 (2.78%)	0.420^e^
Length of hospital stay, days	14.0 (6.0, 26.0)	3.0 (2.0, 5.0)	0.000^c^
ICU admission, n (%)	18 (27.27%)	1 (1.39%)	0.000^e^
Ventilation, n (%)	10 (15.15%)	0 (0.0%)	0.000^e^
Death, n (%)	9 (13.64%)	0 (0.0%)	0.000^e^

Abbreviations: NA, not applicable; ICU, intensive care unit.

^a^ Student’s t-test.

^b^ Chi-squared test.

^c^ *p*<0.0001.

^d^ Mann-Whitney *U* test.

^e^ Fisher exact test.

In univariate analysis, age, sex, PCT, Hb, and WBC were associated with the development of AKI. In the multivariable logistic regression model, elevated PCT levels were independently associated with the development of AKI, with an odds ratio (OR) of 1.480 and a 95% confidence interval (CI) of 1.048–2.091. Elevated WBC counts were also independently associated with AKI, while lowered Hb values were associated with the development of AKI (Table 3).

**TABLE 3:** Regression analysis of factors associated with AKI.

	Univariate analysis		Multivariable analysis	
	OR (95% CI)	*p*-value	OR (95% CI)	*p*-value
Age	1.041 (1.014–1.069)	0.003	1.010 (0.974–1.049)	0.586
Male	1.995 (1.011–3.936)	0.046	1.112 (0.422–2.927)	0.830
PCT	1.978 (1.373–2.851)	0.000	1.480 (1.048–2.091)	0.026
Hb	0.947 (0.927–0.968)	0.000	0.955 (0.927–0.985)	0.003
WBC	1.134 (1.073–1.199)	0.000	1.095 (1.018–1.179)	0.015
Time elapse from bite to hospital	1.019 (0.993–1.046)	0.162		
Hypertension	0.591 (0.231–1.517)	0.274		
Diabetes	2.328 (0.412–13.151)	0.339		

**FIGURE 3:**
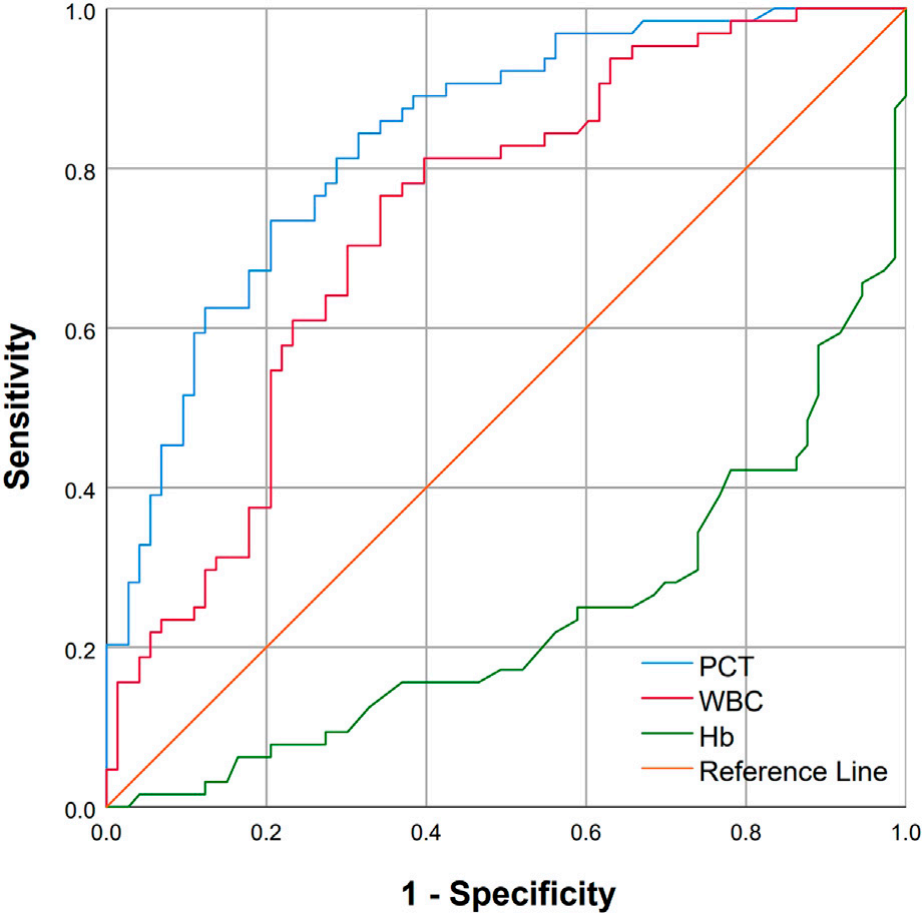
ROC curves of PCT and WBC for predicting AKI among patients with wasp sting. The AUC for PCT was 0.837 (95% CI 0.771–0.902), the AUC for WBC was 0.716 (95% CI 0.652–0.819), and the AUC for Hb was 0.236 (95% CI 0.155–0.317).

Figure 3 shows the predictive value of PCT, WBCs and Hb for predicting the development of AKI in wasp sting patients. The area under the receiver operating characteristic curve (AUROC) for PCT levels on admission for predicting AKI was 0.837 (95% CI 0.771–0.902), which was better than the AUROC for WBC count, 0.716 (95% CI 0.652–0.819). Hb had a poor performance with an AUC of 0.236 (95% CI 0.155–0.317). The optimal PCT cutoff point for AKI was 0.57 μg/L, and the sensitivity and specificity were 73.431% and 79.45%, respectively.

## 4 Discussion

Wasp sting incidents are common worldwide. A large number of published studies have provided information on this public health problem (Xuan et al., 2010; Xie et al., 2013; Witharana et al., 2015; Costa et al., 2018). In this study, we showed elevated serum PCT levels in wasp sting patients. Serum PCT levels had a strong correlation with serum creatinine levels. High PCT levels were independently associated with the development of AKI.

In most cases, wasp stings can only cause local reactions, such as erythema, edema and pain. Usually, those individuals need no medical care and heal spontaneously. However, some people may manifest severe systemic reactions, including AKI, intravascular hemolysis, rhabdomyolysis, coagulopathy, and central nervous system damage (Przybilla and Rueff, 2012). Some individuals may even develop multiple organ dysfunction syndrome (MODS), which can lead to poor clinical outcomes (Vikrant et al., 2005; Grisotto et al., 2006). The hospital mortality rate in this study was 6.52%, which was similar to previous data (5.1%) reported in China (Xie et al., 2013). Among organ dysfunctions, AKI is an important complication and is associated with death (Grisotto et al., 2006; Xuan et al., 2010; Xie et al., 2013). Nearly half of the patients in this study developed AKI, similar to the incidence reported in a study in the Vietnamese population (58.5%) (Xuan et al., 2010), but much higher than the prevalence previously reported in a retrospective multicenter study of 1,091 patients in China (21.0%) (Xie et al., 2013). This may be partly explained by the fact that some of the patients were enrolled from 23 secondary care hospitals, while all patients in the current study came from two tertiary care hospitals.

AKI resulting from wasp stings is characterized by acute tubular necrosis, which can be caused by multiple factors (Grisotto et al., 2006). First, it is believed that renal hypoperfusion plays an essential role in this type of AKI. Components of wasp venom can lead to systemic arterial hypotension and renal vasoconstriction, which result in a reduction in renal blood flow and ischemic renal lesions (Silva et al., 2017). Second, the deposition of myoglobin and hemoglobin in renal tubules was reported by previous studies, suggesting that rhabdomyolysis and hemolysis may also contribute to AKI in wasp sting patients (Vanholder et al., 2000; Grisotto et al., 2006; Daher Ede et al., 2009). Third, venom has a direct toxic effect on kidney tubules (Grisotto et al., 2006). In addition, a previous study reported that age was associated with AKI (Coca, 2010). We also found an association between age and AKI in univariate analysis, but the association was not significant in the multivariable regression analysis.

In the current study, we provided the first evidence of a significant increase in serum PCT levels in the majority of wasp sting patients. As a biomarker of inflammation, PCT production can be stimulated by many disorders (infection, surgery, trauma, shock, burns, *etc.*). The serum PCT level rises rapidly within 2–6 h and peaks within 6–24 h (Vijayan et al., 2017). There are two main mechanisms for the increased production of PCT: 1) direct induction by lipopolysaccharides (LPSs) or other toxic metabolites from microbes and 2) indirectly induced by inflammatory factors such as tumor necrosis factor-alpha (TNF-α), interleukin-6 (IL-6), and interleukin-1 (IL-1) (Vijayan et al., 2017). Previous studies revealed that wasp stings could lead to an increased release of inflammatory cytokines, including serum TNF-α and IL-6 (Xia et al., 2006; Xie et al., 2013; Sun et al., 2018), which can stimulate the production of PCT in wasp sting patients. In addition, many studies have provided evidence that impaired renal function could result in an elevation of serum PCT concentrations (Amour et al., 2008; Heredia-Rodriguez et al., 2016), since renal dysfunction can reduce the elimination of PCT (Meisner et al., 2001).

We found that wasp sting patients who developed AKI had higher PCT levels than non-AKI patients. The association between serum PCT levels and AKI has been reported in previous studies. It was found that PCT can predict the development of AKI in a population with infection (Nie et al., 2013). In another study on critically ill patients, PCT level, as a continuous variable, showed an independent association with AKI (OR, 1.006; 95% CI, 1.000–1.011) (Chun et al., 2019). Moreover, in a retrospective study conducted in a multidisciplinary intensive care unit, a PCT level ≥10 ng/mL was recognized as a significant predictor of AKI in nonseptic patients (OR, 4.430; 95% CI, 1.464–13.399) (Jeeha et al., 2018). In our study, we also found an independent association between PCT levels and AKI. In addition, we found that PCT showed good performance in predicting AKI, with an AUROC of 0.837 (Murphy-Filkins et al., 1996). A PCT level of 0.57 μg/L was the optimal cutoff point for predicting AKI in wasp sting patients.

Our study has several limitations. First, the current study had a relatively small sample size. This makes it difficult to evaluate the relationship between admission PCT levels and hospital mortality. Second, this study was conducted in 2 tertiary hospitals, which may introduce a referral bias because of increased disease severity. In addition, variation in PCT levels during the entire hospital stay was unknown since patients only underwent PCT tests on admission. Sequential blood examinations may be helpful to further evaluate the response to treatment and prognosis of wasp sting patients.

## 5 Conclusion

In this study, we first revealed that serum PCT concentrations increased in wasp sting patients, and PCT levels on admission were independently associated with the development of AKI. Our findings suggest that PCT may be a potential biomarker of AKI in the population of people with wasp stings and may help clinicians recognize subgroups at high risk of developing AKI. However, further multicenter studies are needed to confirm these findings in a larger population.

## Data Availability

The raw data supporting the conclusions of this article will be made available by the authors, without undue reservation.

## References

[B1] Ambarsari C. G. Sindih R. M. Saraswati M. Trihono P. P. (2019). Delayed admission and management of pediatric acute kidney injury and multiple organ dysfunction syndrome in children with multiple wasp stings: A case series. Case Rep. Nephrol. dialysis 9 (3), 137–148. 10.1159/000504043 PMC6902257 31828077

[B2] Amour J. Birenbaum A. Langeron O. Le Manach Y. Bertrand M. Coriat P. (2008). Influence of renal dysfunction on the accuracy of procalcitonin for the diagnosis of postoperative infection after vascular surgery. Crit. care Med. 36 (4), 1147–1154. 10.1097/CCM.0b013e3181692966 18379240

[B3] Bhuiyan M. A. A. Agrawal P. Wadhwaniya S. Li Q. Alonge O. Rahman A. F. (2019). Animal-related injuries and fatalities: evidence from a large-scale population-based cross-sectional survey in rural bangladesh. BMJ open 9 (11), e030039. 10.1136/bmjopen-2019-030039 PMC6830608 31678941

[B4] Chun K. Chung W. Kim A. J. Kim H. Ro H. Chang J. H. (2019). Association between acute kidney injury and serum procalcitonin levels and their diagnostic usefulness in critically ill patients. Sci. Rep. 9 (1), 4777. 10.1038/s41598-019-41291-1 30886220 PMC6423019

[B5] Coca S. G. (2010). Acute kidney injury in elderly persons. Am. J. kidney Dis. 56 (1), 122–131. 10.1053/j.ajkd.2009.12.034 20346560 PMC2902696

[B6] Costa A. G. Chaves B. A. Murta F. L. G. Sachett J. A. G. Sampaio V. S. Silva V. C. (2018). Hymenoptera stings in Brazil: A neglected health threat in amazonas state. Rev. Soc. Bras. Med. Trop. 51 (1), 80–84. 10.1590/0037-8682-0109-2017 29513849

[B7] Daher Ede F. Oliveira R. A. Silva L. S. Silva E. M. Morais T. P. (2009). Acute renal failure following bee stings: case reports. Rev. Soc. Bras. Med. Trop. 42 (2), 209–212. 10.1590/s0037-86822009000200024 19448945

[B8] Grisotto L. S. Mendes G. E. Castro I. Baptista M. A. Alves V. A. Yu L. (2006). Mechanisms of bee venom-induced acute renal failure. Toxicon 48 (1), 44–54. 10.1016/j.toxicon.2006.04.016 16774771

[B9] Heredia-Rodriguez M. Bustamante-Munguira J. Fierro I. Lorenzo M. Jorge-Monjas P. Gomez-Sanchez E. (2016). Procalcitonin cannot be used as a biomarker of infection in heart surgery patients with acute kidney injury. J. Crit. care 33, 233–239. 10.1016/j.jcrc.2016.01.015 26861073

[B10] Herrera C. Leza M. Martínez-López E. (2020). Diversity of compounds in vespa spp. venom and the epidemiology of its sting: A global appraisal. Archives Toxicol. 94 (11), 3609–3627. 10.1007/s00204-020-02859-3 32700166

[B11] Jeeha R. Skinner D. L. De Vasconcellos K. Magula N. P. (2018). Serum procalcitonin levels predict acute kidney injury in critically ill patients. Nephrol. Carlt. Vic. 23 (12), 1090–1095. 10.1111/nep.13174 28967168

[B12] Kellum J. A. Lameire N. Aspelin P. Barsoum R. S. Burdmann E. A. Goldstein S. L. (2012). Kidney disease: improving global outcomes (kdigo) acute kidney injury work group. kdigo clinical practice guideline for acute kidney injury. Kidney Int. Suppl. 2 (1), 1–138.

[B13] Lv Y. Lu L. Yu F. Gao Z. Yuan H. Hu F. (2023). STING deficiency protects against wasp venom-induced acute kidney injury. Inflamm. Res. 72 (7), 1427–1440. 10.1007/s00011-023-01749-5 37326694

[B14] Meisner M. Lohs T. Huettemann E. Schmidt J. Hueller M. Reinhart K. (2001). The plasma elimination rate and urinary secretion of procalcitonin in patients with normal and impaired renal function. Eur. J. Anaesthesiol. 18 (2), 79–87. 10.1046/j.0265-0215.2000.00783.x 11270029

[B15] Murphy-Filkins R. Teres D. Lemeshow S. Hosmer D. W. (1996). Effect of changing patient mix on the performance of an intensive care unit severity-of-illness model: how to distinguish a general from a specialty intensive care unit. Crit. care Med. 24 (12), 1968–1973. 10.1097/00003246-199612000-00007 8968263

[B16] Nie X. Wu B. He Y. Huang X. Dai Z. Miao Q. (2013). Serum procalcitonin predicts development of acute kidney injury in patients with suspected infection. Clin. Chem. laboratory Med. 51 (8), 1655–1661. 10.1515/cclm-2012-0822 23509222

[B17] Przybilla B. Rueff F. (2012). Insect stings: clinical features and management. Dtsch. Arztebl Int. 109 (13), 238–248. 10.3238/arztebl.2012.0238 22532821 PMC3334720

[B18] Silva G. B. D. J. Vasconcelos A. G. J. Rocha A. M. T. Vasconcelos V. R. Barros J. N. Fujishima J. S. (2017). Acute kidney injury complicating bee stings - a review. Rev. do Inst. Med. Trop. Sao Paulo 59, e25. 10.1590/S1678-9946201759025 PMC5459532 28591253

[B19] Sun Y. Yang J. Sun Y. Chen P. Yao W. Meng Z. (2018). Interleukin-6 gene polymorphism and the risk of systemic inflammatory response syndrome caused by wasp sting injury. DNA Cell Biol. 37 (12), 967–972. 10.1089/dna.2018.4156 30265566

[B20] Tang X. Lin L. Yang Y. Y. Huang R. S. Wang B. B. Zhang L. (2022). Development and validation of a model to predict acute kidney injury following wasp stings: A multicentre study. Toxicon 209, 43–49. 10.1016/j.toxicon.2022.02.003 35134424

[B21] Tang X. Wei T. Guan M. Li P. Pu Y. Cheng L. (2023). Phospholipase A(2) induces acute kidney injury by complement mediated mitochondrial apoptosis via TNF-α/NF-κB signaling pathway. Food Chem. Toxicol. 172, 113591. 10.1016/j.fct.2022.113591 36581091

[B22] Thiruventhiran T. Goh B. L. Leong C. L. Cheah P. L. Looi L. M. Tan S. Y. (1999). Acute renal failure following multiple wasp stings. Nephrol. Dial. Transplant. 14 (1), 214–217. 10.1093/ndt/14.1.214 10052513

[B23] Vanholder R. Sever M. S. Erek E. Lameire N. (2000). Rhabdomyolysis. J. Am. Soc. Nephrol. JASN. 11 (8), 1553–1561. 10.1681/ASN.V1181553 10906171

[B24] Vijayan A. L. Vanimaya Ravindran S. Saikant R. Lakshmi S. Kartik R. (2017). Procalcitonin: A promising diagnostic marker for sepsis and antibiotic therapy. J. intensive care 5, 51. 10.1186/s40560-017-0246-8 28794881 PMC5543591

[B25] Vikrant S. Pandey D. Machhan P. Gupta D. Kaushal S. S. Grover N. (2005). Wasp envenomation-induced acute renal failure: A report of three cases. Nephrol. Carlt. Vic. 10 (6), 548–552. 10.1111/j.1440-1797.2005.00482.x 16354236

[B26] Vikrant S. Parashar A. (2017). Wasp venom-induced acute kidney injury: A serious health hazard. Kidney Int. 92 (5), 1288. 10.1016/j.kint.2017.05.035 29055428

[B27] Witharana E. W. Wijesinghe S. K. Pradeepa K. S. Karunaratne W. A. Jayasinghe S. (2015). Bee and wasp stings in Deniyaya; a series of 322 cases. Ceylon Med. J. 60 (1), 5–9. 10.4038/cmj.v60i1.7406 25804910

[B28] Xia C. Y. Zhou J. G. Zhang G. Y. Xie J. P. Liu X. J. (2006). The changes and clinical significance of serum tumor necrosis factor-alpha and endothelin level in bee sting poisoning. Zhonghua nei ke za zhi 45 (7), 579–581. 17074116

[B29] Xie C. Xu S. Ding F. Xie M. Lv J. Yao J. (2013). Clinical features of severe wasp sting patients with dominantly toxic reaction: analysis of 1091 cases. PloS one 8 (12), e83164. 10.1371/journal.pone.0083164 24391743 PMC3877022

[B30] Xuan B. H. Mai H. L. Thi T. X. Thi M. T. Nguyen H. N. Rabenou R. A. (2010). Swarming hornet attacks: shock and acute kidney injury-a large case series from vietnam. Nephrol. Dial. Transpl. 25 (4), 1146–1150. 10.1093/ndt/gfp583 19934093

[B31] Yuan H. Lu L. Gao Z. Hu F. (2020). Risk factors of acute kidney injury induced by multiple wasp stings. Toxicon 182, 1–6. 10.1016/j.toxicon.2020.05.002 32387349

